# Diversity of Secondary Metabolites from Marine *Bacillus* Species: Chemistry and Biological Activity 

**DOI:** 10.3390/md11082846

**Published:** 2013-08-12

**Authors:** Muhammad Abdul Mojid Mondol, Hee Jae Shin, Mohammad Tofazzal Islam

**Affiliations:** 1 School of Science and Technology, Bangladesh Open University, Board Bazar, Gazipur 1705, Bangladesh; E-Mail: mondol_sst@yahoo.com; 2 Marine Natural Products Chemistry Laboratory, Korea Institute of Ocean Science & Technology, Ansan, P.O. Box 29, Seoul 425-600, Korea; 3 Department of Biotechnology, Bangabandhu Sheikh Mujibur Rahman Agricultural University, Gazipur 1706, Bangladesh

**Keywords:** bioactive compounds, macrolactins, detoxification, biosynthesis, carotenoids, biopesticides

## Abstract

Marine *Bacillus* species produce versatile secondary metabolites including lipopeptides, polypeptides, macrolactones, fatty acids, polyketides, and isocoumarins. These structurally diverse compounds exhibit a wide range of biological activities, such as antimicrobial, anticancer, and antialgal activities. Some marine *Bacillus* strains can detoxify heavy metals through reduction processes and have the ability to produce carotenoids. The present article reviews the chemistry and biological activities of secondary metabolites from marine isolates. Side by side, the potential for application of these novel natural products from marine *Bacillus* strains as drugs, pesticides, carotenoids, and tools for the bioremediation of heavy metal toxicity are also discussed.

## 1. Introduction

Microorganisms especially bacteria and fungi are promising sources of structurally diverse and potent bioactive compounds [1,2,3]. Many of these bioactive compounds are used as chemotherapeutic agents for the treatment of human and animal diseases [4,5]. There is a continuing demand for novel bioactive compounds to treat drug-resistant human and animal pathogens [6,7,8], and management of devastating pathogens of crops, which are insensitive or less sensitive to existing chemical pesticides [9,10,11,12].

Marine environments including the subsurface are believed to contain a total of approximately 3.67 × 10^30^ microorganisms [13]. About 70% of the earth’s surface is covered by the ocean representing 80% of life on earth indicating an enormous pool of microbial biodiversity and potential discovery of new natural products [14]. Among diverse microbial species, isolates of marine *Bacillus* belong to phylogenetically and phenogenetically heterogeneous groups of bacteria. They are ubiquitous in the marine environment and can tolerate adverse conditions such as high temperature, pressure, salinity, and pH [15]. Generally, *Bacillus* strains need more nutrition and space to be the fastest growing bacteria for which they compete with other organisms. Due to the diluting effect of the ocean drives, marine organisms produce potent bioactive compounds to fight off their competitors or to escape from micropredation [16,17]. Metabolically marine strains are different from their terrestrial counterparts, and thereby, they may produce unique bioactive compounds, which are not found in their terrestrial counterparts [18,19]. The ability to produce diverse classes of antibiotics by *Bacillus* spp. has been evidence by several genomic studies. For example, the genome sequence of the widely distributed *Bacillus* strains revealed that about 8% of genome is devoted to synthesizing antibiotics [20,21]. Similarly, genome analysis of a marine *B. subtilis* subsp. *spizisenii* strain gtP20b, isolated from the Indian Ocean, indicated the presence of huge number of genes for biosynthesis of secondary metabolites [22]. 

**Figure 1:**
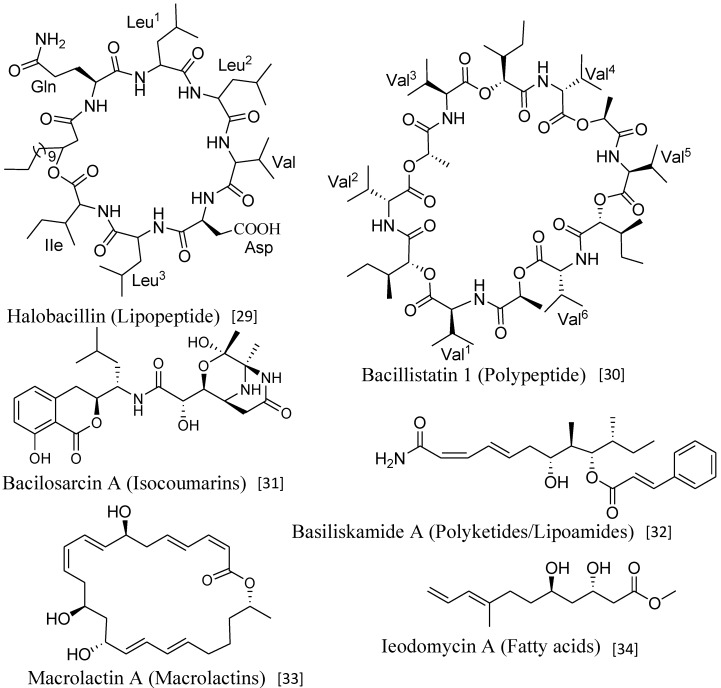
Major classes of secondary metabolites produced by *Bacillus* strains.

Marine *Bacillus* isolates produce structurally diverse classes of secondary metabolites, such as lipopeptides, polypeptides, macrolactones, fatty acids, polyketides, lipoamides, and isocoumarins [23,24] (Figure 1). These structurally versatile compounds exhibit a wide range of biological activities, such as antimicrobial, anticancer, antialgal, and antiperonosporomycetal [23,24]. As *Bacillus* strains rapidly grow in liquid media even under stressful conditions and readily forms resistant spores, it might be useful as an effective biocontrol agent against various phytopathogens [25]. Structures, syntheses, and specific functions of diverse antibiotics produced by *B. subtilis* have elaborately been reviewed [26].

Extensive use of pesticides during crop production and exposure of industrial toxic waste to the environment results in the deposition of heavy metals (Pd, Hg, Cu, Cd, Cr, Co) in soil and water bodies, and this practice can pose serious threats to crop production as well as to the health of all organisms in the aquatic environment. Although trace amount of many heavy metal ions are required for various biochemical activities of all living organisms, higher concentrations of these ions are generally toxic to their cells. Surprisingly, some bacterial species exhibit tolerance and resistance towards high concentrations of heavy metals. Marine strains, in general, thrive under harsh conditions when compared to the most of terrestrial environments, providing them with enormous tolerance, and thus, they are often considered as potential candidates for the detoxification of heavy metals [27]. Most of the toxic heavy metals are reduced rather than oxidized by native microbes, with a few exceptions, since the reduced forms are, generally, less toxic. Bacterial tolerance to heavy metals generally follows the pattern of efflux, accumulation, complexation, and reduction [28]. 

Nowadays, food growers heavily rely on chemical pesticides to prevent or control diseases in their crop plants. Deposition of these pesticide residues in food, soil, and water bodies is imposing enormous threats to human health, environment, and ecosystem [35]. Consequently, there is an increasing demand from consumers and environment conservationists to replace chemical pesticides with natural, environment-friendly antagonistic microorganisms with novel mode of action for sustainable production of crops [11,12].

As marine *Bacillus* isolates produce diverse bioactive secondary metabolites with novel modes of action, they may have great potential for the development of effective management strategies to combat human, animal and phytopathogens in biorational manners. Several general reviews on bioactive secondary metabolites from *Bacillus* isolates have been published [23,26], however, no report has so far been published on bioactive compounds from marine *Bacillus* species. This article comprehensively reviews the chemistry and biological activities of diverse secondary metabolites from marine *Bacillus* species, and discusses the potentials of these natural products for the development of effective drugs, agrochemicals, carotenoids, as well as tools for the bioremediation of environmental pollution due to heavy metal contamination.

## 2. Bioactive Compounds

### 2.1. Lipopeptides

Cyclic lipopeptides (cLPs) are versatile metabolites produced by a variety of bacterial genera. They are composed of a short cyclic oligopeptide linked to a fatty acid tail, and exhibit potent surfactant properties [36]. cLPs are produced nonribosomally on large NRPS (nonribosomal peptide synthetase)—PKS (polyketide synthetase) hybrid synthetases. Endospore-forming rhizobacterium (terrestrial) *B. subtilis* produces varieties of antimicrobial peptides that are either ribosomally synthesized and post-translationally modified (e.g., lantibiotics and lantibiotic-like peptides), or nonribosomally generated. Ribosomally and nonribosomally synthesized peptides by terrestrial *Bacillus* spp. are elaborately reviewed by Stein [26]. cLPs have received considerable attention for their antibiotic activities against a range of human- and plant-pathogenic organisms, including enveloped viruses, mycoplasmas, trypanosomes, bacteria, fungi, and peronosporomycetes [37]. 

**Figure 2:**
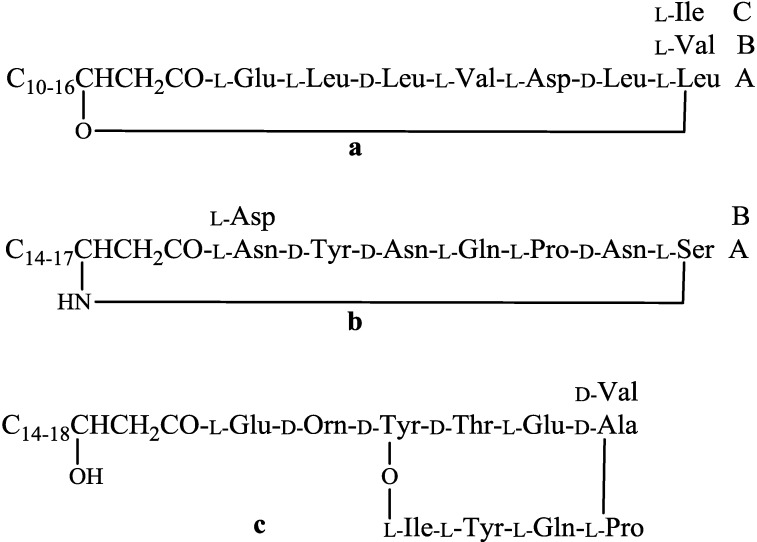
General structures of surfactin (**a**); iturin (**b**) and fengycin (**c**).

A large proportion of the secondary metabolites produced by the *Bacillus* isolates are cyclic lipopeptides belonging to three families: iturins, fengycins, and surfactins [38]. The chemical structure of c-LPs have a peptide backbone composed of seven (iturins and surfactins) or 10 (fengycins) amino acids connected to a β-hydroxy (fengycins and surfactins) or β-amino (iturins) fatty acid, which may vary from C-10 to C-16 for surfactins, C-14 to C-17 for iturins, and C-14 to C-18 for fengycins. Each lipopeptide family can be subdivided again based on position of specific amino acid in the peptide ring. For example, the fengycin family is subdivided into fengycin A and fengycin B, where D-alanine and D-valine is present in the sixth position, respectively. Due to difference in length, branching, and saturation of acyl chain, homologues are formed within each subdivision of cLPs (Figure 2). Based on the comparison of retention times and molecular masses with those of known compounds, all three families of lipopeptide antibiotics may be assigned using LC-MS with specific elution programs [39].

A cLPs producer, *B. amyloliquefaciens* strain GA1 was initially isolated from strawberry culture. To identify gene fragments responsible for synthesis of cLPs, partial DNA sequencing of strain GA1 has been done. Analysis of sequencing data revealed that fourteen gene fragments with homology toward gene clusters were involved in the synthesis of cLPs [38]. Out of fourteen, in eleven gene fragments, operons *srf* and *fen* had 80%–96% and 41%–92% amino acid identity directing the synthesis of surfactin and fengycin, respectively [38]. Further analysis of sequencing data revealed that three gene fragments have been involved in directing the synthesis of an iturin lipopeptide in the strain FZB42 and have 48%–82% amino acid similarity with the itu*DABC* operon encoding the iturin A synthetase in *B. subtilis* RB14 [40]. 

**Figure 3:**
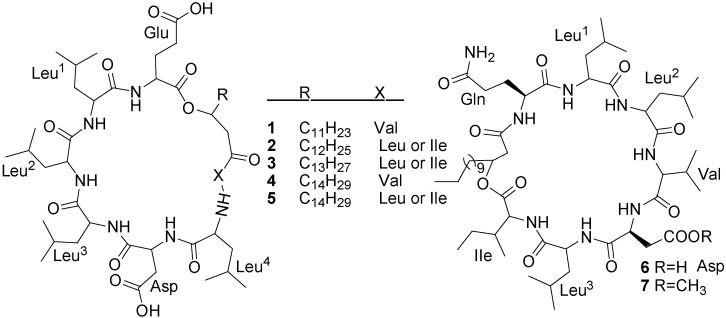
Structures of surfactin analogs (**1**–**5**) and halobacillins (**6** and **7**).

Five surfactin analogs (**1**–**5**) (Figure 3) have been isolated from the culture broth of a *Bacillus pumilus* (SP21) through bioassay-guided fractionation [41]. The producing strain was isolated from a sediment sample collected from the Bahamas. Compounds **1**–**4** showed selectively good inhibitory activity against *S. aureus*, *P. vulgaris*, and *E. faecalis* (6.5 to 25 µg/mL), but not against *P. aeruginosa* [41].

Halobacillin (**6**) and methyl halobacillin (**7**) (Figure 3), two cyclic acylpeptides have been isolated from the culture broth of a *Bacillus* sp. CND-914. The strain CND-914 was obtained from a deep-sea sediment core taken at near Guaymas Basins in Mexico [29]. Halobacillin, the first novel acylpeptide of the iturin class similar to surfactin, is one of the most effective known biosurfactants [42]. The major difference between halobacillin and surfactin is the replacement of glutamic acid of surfactin with a glutamine in halobacillin. Halobacillin inhibits the growth of human colon tumor cells (HCT-116) with an IC_50_ of 0.98 µg/mL but it does not exhibit antimicrobial activity like surfactin (Table 1). 

**Figure 4:**
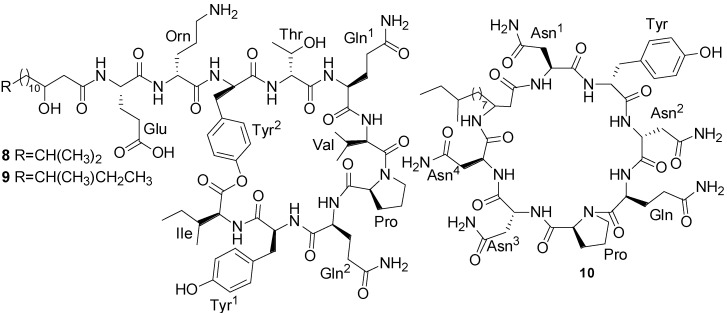
Structures of fengycins (**8** and **9**) and mojavensin (**10**).

Three lipopeptides (**8**–**10**) (Figure 4), iso-C16 fengycin B (**8**), anteiso-C17 fengycin B (**9**) and a new iturinic lipopeptide, mojavensin A (**10**), were obtained by bioactivity-guided fractionation from the fermentation broth of *B. mojavensis* B0621A, which was isolated from the mantle of a pearl oyster *P. martensii* collected from Weizhou Island in the South China Sea [43]. These lipopeptides displayed dose-dependent antifungal activity against a broad spectra of phytopathogens as well as being weakly antagonistic to *S. aureus*. Moreover, they all displayed cytotoxic activities against the human leukemia (HL-60) cell line with IC_50_ values of 100, 100, and 1.6 µM, respectively [43].

**Table 1:** List of some important bioactive compounds isolated from marine strains.

Compounds	Producing strains	Test organisms/cell lines	Inhibitory concentrations	Nature of bioactivities	Ref.
Halobacillin (**6**)	*Bacillus* sp. CND-914	Human HCT-116 cancer cells	0.98 µg/mL (IC_50_)	Anticancer	[29]
Mixirin (**11**)	*Bacillus* sp.	Human HCT-116 cancer cells	0.68 µg/mL (IC_50_)	Anticancer	[44]
Bogorol A (**15**)	*Bacillus* sp.	MRSA	2 µg/mL (MIC)	Antibacterial	[45]
Loloatin B (**18**)	*Bacillus* sp.	MRSA, VRE	1–2 µg/mL (MIC)	Antibacterial	[46]
Bacillistatins 1 (**19**) and 2 (**20**)	*B. sil*v*estris*	Human cancer cell line	10^−4^–10^−5^ µg/mL (GI_50_)	Anticancer	[30]
Bacillamide (**27**)	*Bacillus* sp.	*C*. *polykrikoides*	LC_50_ after 6 h: 3.2 µg/mL	Antialgal	[47]
Bacilosarcin A (**35**)	*B. subtilis*	Barnyard millet sprouts	82% inhibition at 50 µM	Plant growth regulator	[31]
Macrolactin S (**69**)	*B. amyloliquefaciens*	*E. coli* and *S. aureus*	0.3 and 0.1 µg/mL (MIC)	Antibacterial	[48]
Macrolactin V (**86**)	*B. amyloliquefaciens*	*E. coli*, *B. subtilis* and *S. aureus*	0.1 µg/mL (MIC)	Antibacterial	[48]
Basiliskamides A (**21**) and B (**22**)	*B. laterosporus*	*C. albicans* and *A. fumigatus*	1.0 and 3.1 µg/mL 2.5 and 5.0 µg/mL	Antifungal	[32]

**Figure 5:**
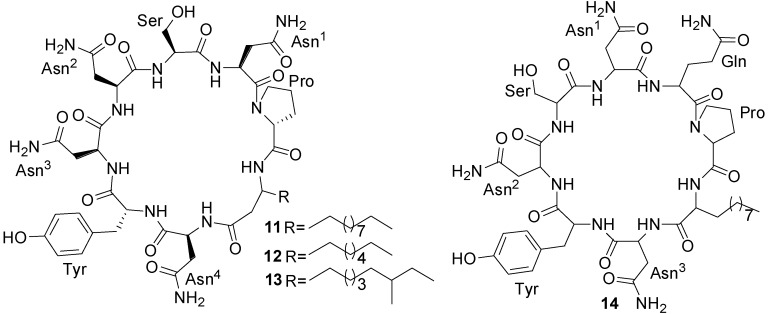
Structures of mixirins A–C (**11**–**13**) and marihysin A (**14**).

Three new cyclic acylpeptides named mixirins A–C (**11**–**13**) (Figure 5) belonging to the iturin class have been isolated from marine bacterium *Bacillus* sp. [44]. This isolate was obtained from sea mud near the Arctic pole. Mixirins A, B, and C inhibited the growth of human colon tumor cells (HCT-116) with IC_50_ of 0.68, 1.6, 1.3 µg/mL, respectively (Table 1) [44].

A new cyclic lipopeptide, marihysin A (**14**) (Figure 5) was isolated from the fermentation broth of the marine *B. marinus* B-9987 isolated from the tissues of rhizophere of *Suaeda salsa* in the intertidal zone of the Bohai Bay, China, which exhibited a broad-spectrum activity against plant pathogens with minimum inhibitory concentrations (MICs) of 100–200 µg/mL [49].

### 2.2. Polypeptides

Nonribosomal peptides (NRPs) are synthesized by large multimodular nonribosomal peptide-synthetase (NRPS) by elongation of activated monomers of amino acid building blocks. NRPSs are organized in modules responsible for the incorporation of a specific amino acid in polypeptide chain by three successive steps: adenylation, thiolation, and condensation [50]. Two types of polypeptides are generally produced by *Bacillus* strains are linear and desipeptides. 

**Figure 6:**
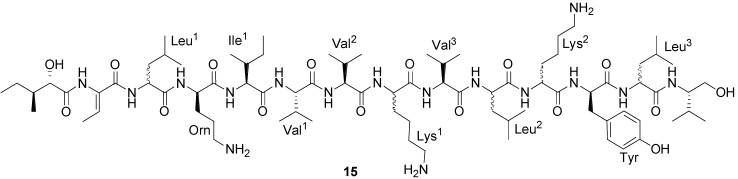
Structure of bogorol A (**15**).

Bogorol A (**15**) (Figure 6), a novel peptide antibiotic has been obtained from the culture broth of a marine *Bacillus* sp. isolated from a tropical reef habitat in Papua New Guinea [51]. Bogorol A illustrates a new structural template for “cationic peptide antibiotics”, and it showed good activity against methicillin-resistant *S. aureus* (MRSA) (MIC 2 µg/mL), vancomycin-resistant enterococcus (VRE) (MIC 10 µg/mL) and moderate activity against *E. coli* (MIC 35 µg/mL), and no activity against *S. maltophilia* (>200 µg/mL).

**Figure 7:**
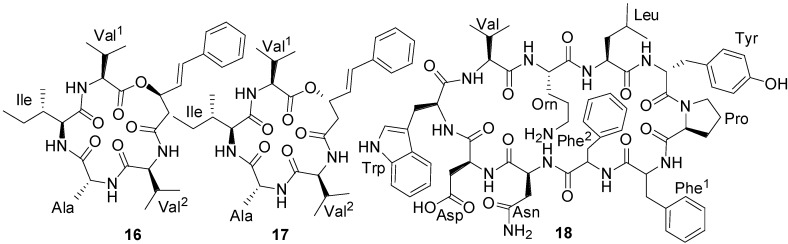
Structures of turnagainolides A and B (**16** and **17**), and loloatin B (**18**).

Two new cyclic depsipeptides, turnagainolides A (**16**) and B (**17**) (Figure 7), have been isolated from laboratory cultures of a marine strain RJA2194 [51]. This strain was isolated from a sediment sample collected near Turnagain Island and identified as a *Bacillus* species. The structures of **16** and **17** are representative of epimers at the site of macrolactonization. Turnagainolide B (**17**) showed activity in a SHIP1 activation assay [51].

Loloatin B (**18**) (Figure 7) is a novel cyclic decapeptide antibiotic with potent antimicrobial activity against Gram-positive bacteria produced by a *Bacillus* sp. which was isolated from the tissues of a marine worm [46]. In screening tests for antimicrobial activity, loloatin B inhibited the growth of MRSA, VRE, and penicillin resistant *S. pneumoniae* with MICs of 1–2 µg/mL. 

**Figure 8:**
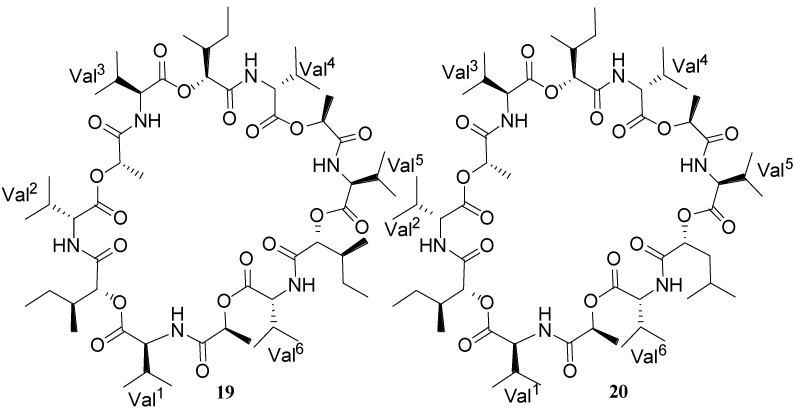
Structures of bacillistatins 1 (**19**) and 2 (**20**).

Two new cyclodepsipeptides designated as bacillistatins 1 (**19**) and 2 (**20**) (Figure 8) have been isolated from the culture broth of *Bacillus sil*v*estris* that was obtained from a Pacific Ocean (southern Chile) crab [30]. Each 12-unit cyclodepsipeptide (**19** and **20**) strongly inhibited the growth of a human cancer cell line panel, with GI_50_’s of 10^−4^–10^−5^ µg/mL, and each compound was determined to be active against antibiotic-resistant *S. pneumoniae*.

### 2.3. Polyketides/Lipoamides

Polyketides are extremely large classes of secondary metabolites that are assembled from simple acyl-coenzyme A and form the basis of numerous pharmaceuticals, agrochemicals, and veterinary agents. Polyketides are biosynthesized by polyfunctional megasynthases organized into repeated functional units known as modules. The modular megaproteins responsible for polyketide biosynthesis are known as polyketide synthases (PKSs) [50,52]. Due to their mechanism of versatile assemblage, the polyketides exhibit remarkable diversity both in terms of structure and biological activities. 

**Figure 9:**
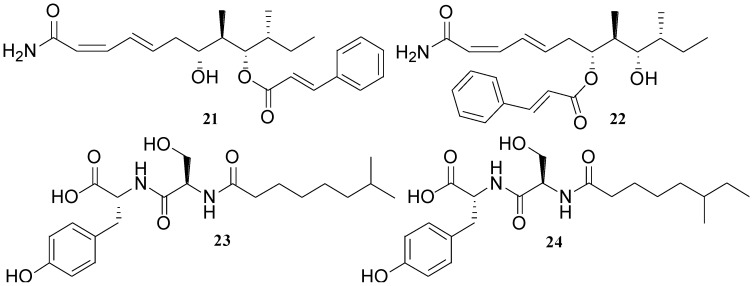
Structures of basiliskamides (**21** and **22**) and tupuseleiamides (**23** and **24**).

A *B. laterosporus* isolate, obtained from coastal waters of Papua New Guinea, has been shown to produce some novel metabolites such as basiliskamide A (**21**), basiliskamide B (**22**), tupuseleiamide A (**23**), and tupusleiamide B (**24**) [32] (Figure 9). Compound **22** was simply a regioisomer of **21**, and **24** was a regioisomer of **23**. The observed MIC values of **21** and **22** against *C. albicans* and *A. fumigatus* were 1.0 and 3.1 µg/mL, and 2.5 and 5.0 µg/mL, respectively.

**Figure 10:**
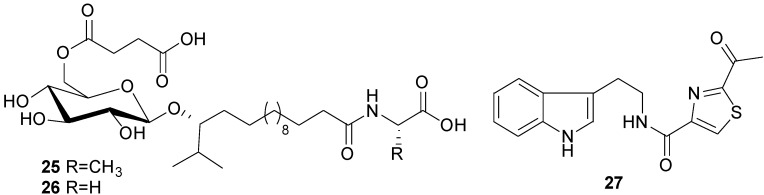
Structures of ieodoglucomides A and B (**25** and **26**), and bacillamide (**27**).

Two unique polyketides, ieodoglucomides A (**25**) and B (**26**) (Figure 10) were isolated from a marine-derived bacterium *B. licheniformis* [53]. This bacterium was isolated from a sediment sample collected from Ieodo in Republic of Korea’s southern reef. Compounds **25** and **26** displayed moderate *in vitro* antimicrobial activity against both Gram-positive and Gram-negative pathogenic bacteria (MIC 8–32 µg/mL). Furthermore, ieodoglucomide B (**26**) exhibited cytotoxic activity against lung cancer and stomach cancer cell lines with GI_50_ values of 25.18 and 17.78 µg/mL, respectively [52]. 

Bacillamide (**27**) (Figure 10) was isolated from *Bacillus* sp. SY-1, which was obtained from seawater during the blooming period of *C. polykrikoides* in Masan Bay [47]. Bacillamide (**27**) showed significant algicidal activity against *C*. *polykrikoides* (LC_50_ after 6 h: 3.2 µg/mL) and selective activity against dinoflagellates and raphidophytes. However, **27** showed neither algicidal activity against microalgae of other phyla such as diatom, green algae, and cyanobacteria, nor growth inhibitory effects against bacteria, fungi, or yeast. Therefore, **27** might be considered as a useful algicidal agent for regulating the blooms of harmful dinoflagellate species such as *C*. *polykrikoides.*

**Figure 11:**
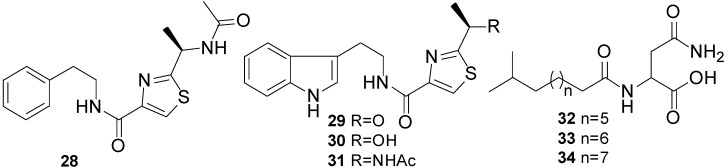
Structures of thiazole alkaloids (**28**–**31**) and lipoamides A–C (**32**–**34**).

A novel thiazole alkaloid, neobacillamide A (**28**) together with three known related bacillamides A–C (**29**–**31**), were isolated from the bacterium *B. atrophaeus*, which was associated with the South China Sea sponge *Dysidea avara* [54] (Figure 11). It is interesting to note that all bacillamides A–C (**29**–**31**) contain a common tryptamine moiety in their molecules while in **28** the amine portion is replaced by a phenethylamine. Neobacillamide A was the first member of *Bacillus* thiazole alkaloids, which has been determined to contain a phenethylamine moiety. Compounds **28** and **31** were evaluated for their inhibitory activity against HL60 human leukemia cells and A549 human lung cancer cells but both compounds were found inactive.

Three new compounds named lipoamides A–C (**32**–**34**) (Figure 11) have been isolated from the culture broth of a sediment *B. pumilus* (SP21) [41]. Lipoamide A (**32**) showed weak antibacterial activity against *S. aureus* and *P. aeruginosa* (MIC > 100 µg/mL).

### 2.4. Isocoumarins

Isocoumarin-type metabolites from microorganisms are characterized by the amino-containing substituent, which is presumably derived from leucine, at the 3-position in the dihydrocoumarin core. There have been several such isocoumarin compounds as baciphelacin [55], amicoumacins [56,57,58,59], and xenocoumacins [60]. These compounds possess the common chromophore, 3,4-dihydro-8-hydroxyisocoumarin in their structures and many of them are produced by the genus *Bacillus*. 

**Figure 12:**
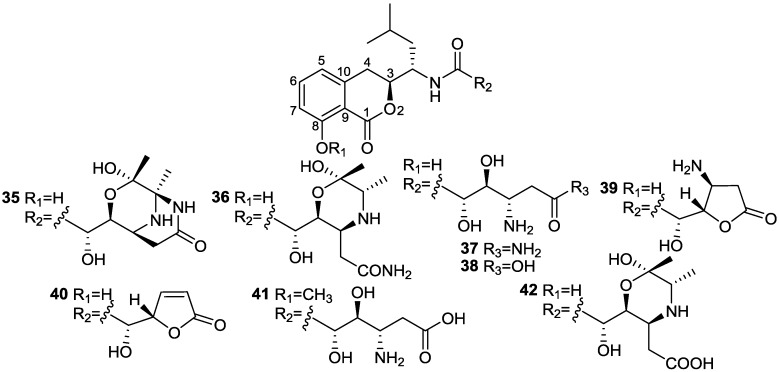
Structures of isocoumarins (**35**–**42**).

Two novel isocoumarins, bacilosarcins A (**35**) and B (**36**), and three known isocoumarins, amicoumacins A (**37**), B (**38**), and C (**39**), were isolated from the culture broth of a marine-derived bacterium *B. subtilis* TP-B0611 [31] (Figure 12). The strain B0611 was isolated from the intestine of a sardine (*S. melanosticta*) collected in Toyama Bay in Japan. Compound **35** possesses an unprecedented 3-oxa-6,9-diazabicyclo[3.3.1]nonane ring system, where **36** has a 2-hydroxymorpholine moiety which is rare in nature. Compound **35** showed 82% inhibition at 50 µM against growth of barnyard millet sprouts and on the contrary **36** showed very weak activity at the same concentration. Of particular interest, amicoumacin A (**37**) showed more potent activity than **35**. On the basis of biogenetic consideration, it is reasonable to assume that compound **35** was derived from amicoumacin A (**37**), forming a biacetyl equivalent C_4_ unit and two water molecules. This may imply that **35** behaves as a prodrug of **37** in plant cells. The activity levels shown by **35** and **37** are higher than that of herbimycin A, a potent herbicidal compound from *Streptomyces* [61], suggesting that they may be lead molecules for plant growth regulators.

**Figure 13:**
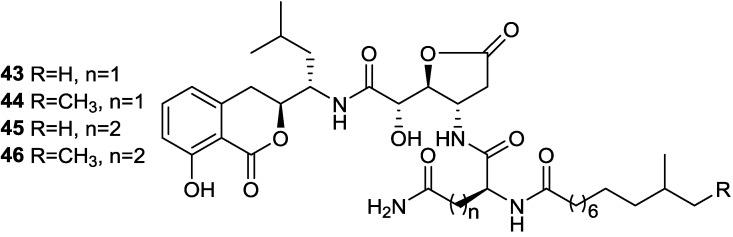
Structures of lipoamicoumacins A–D (**43**–**46**).

Six known analogs (**36**–**41**), one new bacisarcin C (**42**) (Figure 12) and four novel amicoumacins, lipoamicoumacins A–D (**43**–**46**) (Figure 13) were isolated from the culture broth of a marine-derived bacterium, *B. subtilis* B1779 [62]. The strain B1779 was isolated from a marine sediment sample, which was collected from the Red Sea in April 2010. All isolated compounds were evaluated for cytotoxicity and antibacterial activities. Only compounds **36** and **37**, which have an amide functional group (-NH_2_) exhibited cytotoxicity against HeLa cells with IC50 values of 33.60 and 4.32 µM, respectively, indicating that the amide group of amicoumacin plays a critical role in cytotoxicity. This was further supported by a comparison of cytotoxicity between compounds **36** and **42** and between **37** and **38**. A similar antibacterial effect of amide functional group has been shown among amicoumacins [62].

### 2.5. Fatty Acids

Bacteria are known to synthesize fatty acids via the classic fatty acid synthase (FAS) pathway with chain length ranging from C_12_ to C_19_ [63,64]. Fatty acid from acetyl-CoA and malonyl-CoA precursors through the action of enzymes called fatty acid synthases (FAS). 

Methyl-branched fatty acids with methyl groups at even carbon atoms (methyl substituents on carbon 2, 4, 6) occur in several organisms, and originate from the selective incorporation of methylmalonyl-CoA by fatty acid synthases [65]. The *iso*-*anteiso* branching in the novel methyl-branched fatty acids is most probably derived from leucine and isoleucine followed by a series of elongations with malonyl-CoA. At either the last or penultimate elongation step methylmalonyl-CoA seems to be selectively incorporated by one of the fatty acid synthesizing enzymes from the bacterium resulting in the methyl-branched fatty acids. Whether this is a known or unknown, methyl-branched fatty acid synthase in bacteria is, as of yet, a matter of speculation.

**Figure 14:**
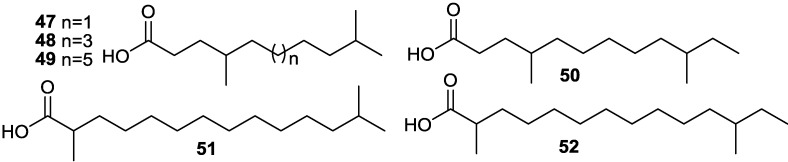
Structures of *iso*-*anteiso* fatty acids (**47**–**52**).

A series of novel *iso*-*anteiso* fatty acids (**47**–**52**) (Figure 14) with chain lengths between C_11_ and C_19_ and an interesting series of linear alkylbenzene fatty acids with chain lengths between C_10_ and C_14_ were produced by a halophilic *Bacillus* sp. [66]. The biological activity of these novel fatty acids has not yet been reported. 

**Figure 15:**
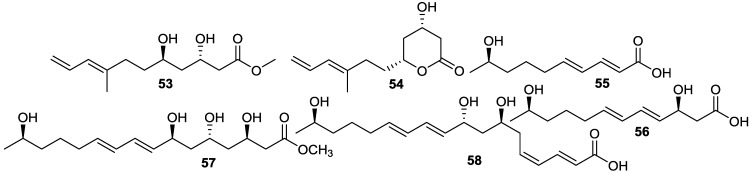
Structures of unsaturated fatty acids (**53**–**58**).

Bioassay-guided isolation of the EtOAc extract of a marine *Bacillus* sp. 09ID194 cultured in modified Bennett’s broth medium, yielded six new unsaturated hydroxy fatty acids, named ieodomycins A–D (**53**–**56**) [34] and linieodolides A and B (**57** and **58**) [67] (Figure 15). The producing strain was isolated from a sediment sample collected from Ieodo, Republic of Korea’s southern reef. Compounds **53**–**58** exhibited antimicrobial activity against *B. subtilis* and *E. coli* with MICs of 32–64 µg/mL, but showed weak growth inhibition against the yeast, *S. cerevisiae*, with an MIC of 128–256 µg/mL.

### 2.6. Macrolactins

The macrolactins are polyene cyclic macrolactones consisting of 24-membered ring lactones with modifications such as the attachment of glucose β-pyranoside, and they may also occur as linear analogs [33]. The macrolactin carbon skeleton contains three separate diene structure elements in a 24-membered lactone ring. The macrolactin class of macrolactones is mainly produced by both terrestrial and marine strains [23]. 

In the genome of *B. amyloliquefaciens* FZB42, a plant-root-colonizing environmental strain with the ability to stimulate plant growth and to suppress soil-borne plant pathogens in the rhizosphere [68,69], three PKS operons altogether about 196,340 pb were located at sites approximately 1.4 Mbp (*pks*2), 1.7 Mbp (*pks*1), and 2.3 Mbp (*pks*3) clockwise from the origin of replication in the genome which is 3916 kb in size [70,71]. These three-gene clusters show a modular organization which is typical for type I PKS systems, indicating that the strain FZB42 has the biosynthetic machinery for the production of at least three different kinds of polyketides. *pks*1 and *pks*3 have been attributed to the production of bacillaene and difficidin/oxydifficidin, respectively, where the *pks*2 is involved in macrolactins biosynthesis. The macrolactone rings of macrolactins are formed via cyclization of polyketide chains assembled by PKS type-I enzymes that perform repetitive decarboxylative condensations of carboxylic acids with an activated carboxylic acid starter unit [72]. 

**Figure 16:**
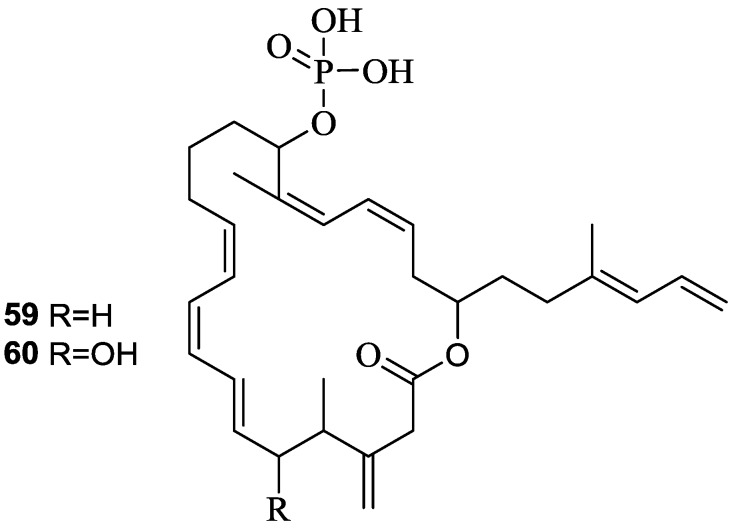
Structures of difficidin (**59**) and oxidifficidin (**60**).

Difficidin (**59**) and oxidifficidin (**60**) (Figure 16), detected in the culture broth of *B. amyloliquefaciens* (FZB42) are highly unsaturated 22-membered macrocyclic polyene lactone phosphate esters with broad-spectrum of antibacterial activity [73]. Difficidin (**59**) has recently shown promising suppressive activity against the enterobacterium *Erwinia amylovara*, a devastating plant pathogen which causes necrotrophic fire blight disease affecting apple, pear, and other rosaceous plants [74].

The primary mode of action of difficidin (**59**) is the inhibition of protein synthesis. It has proven to be highly bactericidal to both growing and stationary phase cultures and inhibited protein synthesis more rapidly than RNA, DNA, or cell-wall synthesis in growing cells [75].

At least 32 macrolactins have been characterized so far including macrolactins A–Z, 7-*O*-succinyl macrolactin A, 7-*O*-succinyl macrolactin F, 7-*O*-malonyl macrolactin A, and three ether-containing macrolactin A. Most of them were produced by marine sediment isolates and few of them were by soil isolates [76,77]. 

**Figure 17:**
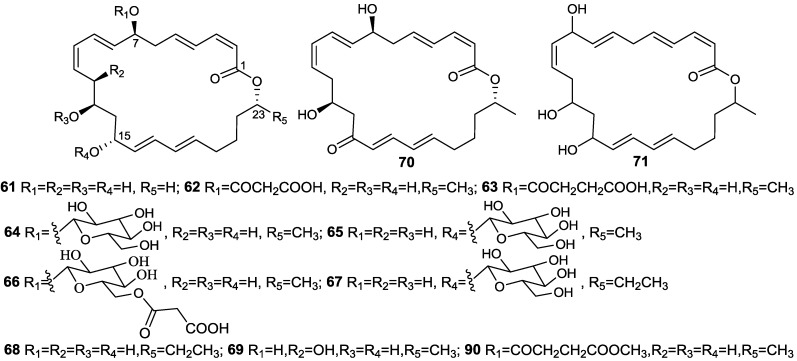
Structures of 24-membered macrolactin A (**61**) and its derivatives (**62**–**71** and **90**).

**Figure 18:**
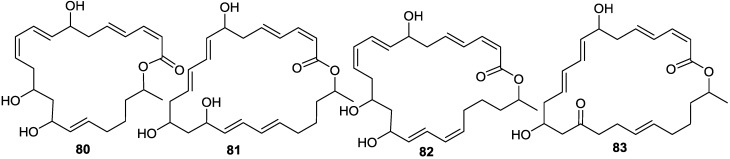
Structures of 22- (**80**) and 24-membered (**81** and **82**) including keto (**83**) macrolactins.

The first macrolactin family, including macrolactins A–F (**61**, **64**–**66**, **70**, **74**) (Figure 17 and Figure 18), as well as the open-chain macrolactinic and isomacrolactinic acid, were isolated from an unclassified deep-sea sediment bacterium [33]. Macrolactin A was of particular interest because of its biological activities. It shows selective antibacterial activity against *S. aureus* and *B. subtilis* at a concentration of 5 and 20 µg/disc, respectively, as well as the ability to inhibit B16-F10 murine melanoma cancer cells in *in vitro* assays, as well as mammalian *Herpes simplex* viruses. In addition, it protects lymphoblast cells against HIV by inhibiting virus replication [33]. 

**Figure 19:**
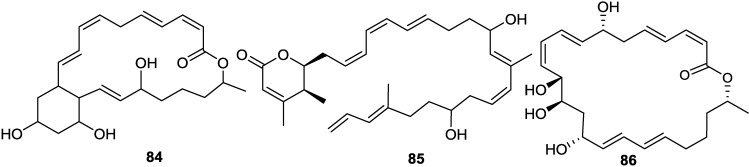
Structures of bicyclic (**84**) and polyene (**85**) macrolactins, and 12-hydroxy macrolactin A (**86**).

Macrolactins G–M (**71**, **80**–**84**, **68**) (Figure 17, Figure 18, Figure 19) were produced by a *Bacillus* sp. PP19-H3, which was isolated from a marine macroalga, *Schizymenia dubyi*, collected at the Omaezaki coast of Shizuoka prefecture in Japan [78]. These macrolactins include a 22-membered ring (**80**) or bicyclic lactone (**84**) in addition to their geometric isomers of macrolactins A and F. These macrolactins were more active against *S. aureus* (MIC 5–10 µg/mL) than *B. subtilis* (MIC 60 µg/mL), whereas macrolactin K (**83**), containing keto group (C=O) at C-15 showed very weak antibacterial activity against both tested pathogens (MIC > 100 µg/mL), indicating that the -OH group at C-15 may play an important role in the antibacterial activity of macrolactins [78].

**Figure 20:**
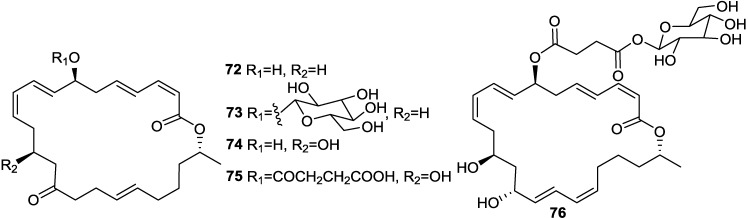
Structures of keto (**72**–**75**) and ester (**76**) macrolactins.

**Figure 21:**
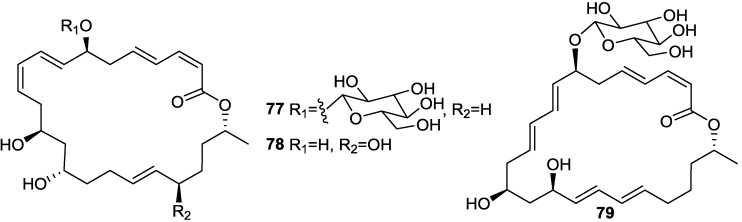
Structures of glycosylated (**77** and **79**) and a 24-membered (**78**) macrolactins.

With a view to find out peptide deformylase inhibitors, four glycosylated macrolactins O–R (**73**, **67**, **77**, and **79**) (Figure 17, Figure 20 and Figure 21) were isolated from the liquid cultures of *Bacillus* sp. AH159-1 [72]. Macrolactins O–R inhibited *S. aureus* peptide deformylase (PDF) in dose-dependent manners with IC_50_ (µM) values of 53.5, 57.7, 12.1, and 61.5, respectively. All these compounds also inhibited bacterial growth against *E. coli* with an MIC of 100 µg/mL [77]. Macrolactin N (**72**) (Figure 18) also inhibited *S. aureus*’s peptide deformylase (PDF) in a similar fashion to macrolactins O–R [79].

A new macrolactin T (**78**) and a new polyene δ-lactone macrolactin U (**85**) (Figure 19 and Figure 21), along with known macrolactins A, B, D, O and S (**69**) were isolated from the culture broth of the bacterium *B. marinus*, which was isolated from *Suaeda salsa* collected on the coastline of the Bohai Sea of China [80]. Macrolactins T and B exhibited MIC activity toward *A. solani*, *P. oryzae*, and *S. aureus*, at concentrations of 0.8, 2.8, 5.5 and 7.5, 20.1, 4.5, respectively [80].

Macrolactins V (**86**) and S (**69**) (Figure 17 and Figure 21) were isolated from the culture broth of a marine bacterium, *B. amyloliquefaciens* SCSIO 00856, which was isolated from a South China Sea gorgonian, *Junceella juncea* [48]. Macrolactin V (**86**) exhibited potent antibacterial activity against *E. coli*, *B. subtilis* and *S. aureus* with an MIC value of 0.1 µg/mL, and no activity against *B. thuringiensis*. Macrolactin S (**69**) showed strong antibacterial activity against both *E. coli* and *S. aureus* with MIC values of 0.3 and 0.1 µg/mL, respectively, but weak activity against *B. subtilis* (MIC 100 µg/mL), which indicated that the configuration of 7-OH may affect the antibacterial activity of the epimers **86** and **69** [48]. 

7-*O-*succinyl macrolactin A (**63**) and 7-*O*-succinyl macrolactin F (**75**) (Figure 17 and Figure 18) were isolated from a marine sediment *Bacillus* sp. Sc026 [81], and 7-*O*-malonyl macrolactin A (**62**) (Figure 17) from a *B. subtilis* [82]. The bacterium (Sc026) was isolated from marine sediment collected from nearby Sichang Island (at a 15-m depth), Chonburi, Thailand. **63** and **75** were tested in an agar diffusion assay at concentration of 50 and 100 µg/disk, respectively. The inhibition zones of **63** and **75** against *B. subtilis* and *S. aureus* were 10 and 9, and 24 and 8 mm, respectively. The minimum restrictive concentrations (MRCs) of **62** were between 1 and 64 µg/mL for *S. aureus* and MRSA strains, and between 0.06 and 4 µg/mL for *E. faecalis* and clinical isolates, VRAS (vancomycin-resistant/ampicillin-sensitive) E305 and VRAR (vancomycin-resistant/ampicillin-resistant) E315 [82]. 

**Figure 22:**
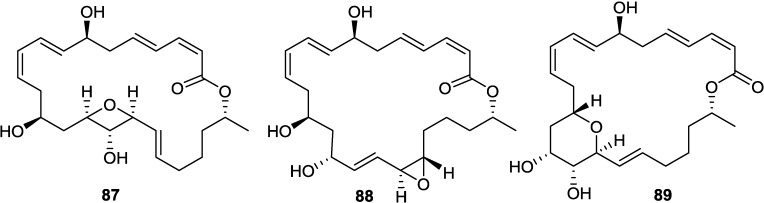
Structures of ether-containing macrolactins (**87**–**89**).

**Figure 23:**
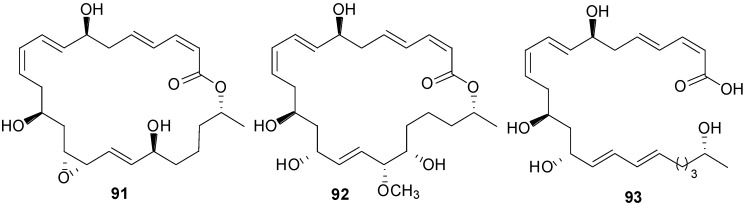
Structures of ether (**91**) and methoxy (**92**) containing macrolactins and macrolactinic acid (**93**).

Macrolactins W (**76**), Y (**91**), Z (**92**), X (**90**), macrolactinic acid (**93**) and three ether-containing unique macrolactins (**87**–**89**) (Figure 17, Figure 18, Figure 22, and Figure 23) were isolated from a sediment *Bacillus* sp. 09ID194 [67,83,84]. Interestingly, these compounds were produced by this strain only in low salinity culture medium (12 g/L) but not in high salinity culture medium (32 g/L). These compounds showed MIC against pathogenic bacteria at a concentration range of 8–64 µg/mL. The position of ether group was important for the antimicrobial activity of these ether-containing macrolactins (**87**–**89**) [84]. 

## 3. Detoxification of Heavy Metals

Chromium (Cr) is one of the major causes of environmental contamination by heavy metals. The toxicity of chromium (Cr) lies in its oxidative states [85]. The higher oxidation states impose more toxicity (10–100 times) than the lower oxidation states [86]. Nutritionally, small amount of Cr(III) is essential for balanced human and animal diet for preventing adverse effects caused by glucose and lipid metabolisms [87,88]. Swallowing large amounts of Cr(III) may pose threats to human health [89,90]. Hexavalent Cr (Cr^6^^+^) is extremely carcinogen and may be the cause of death after ingestion of a large dose [91]. The tolerable intake level of Cr(VI) and Cr(III) are 1 and 8 µg/L for freshwater life, 1 and 50 µg /L for marine life, and 8 and 5 µg/L for irrigation water, respectively [92,93].

A marine isolate, *B. licheniformis*, can reduce 10–500 mg/L of Cr(VI) to Cr(III) within 24–72 h in liquid medium [94]. When this strain was cultured in the liquid medium, it produces chromium reductase indicating that probably Cr(VI) may be reduced to Cr(III) by enzymatic activity. This strain also secretes an extracellular surface-active agent (biosurfactant) in the medium, which provides tolerance to the cells towards hexavalent chromium and protects the cells from oxidative stress. 

The oxidation of soluble manganese(II) to insoluble Mn(III, IV) oxide plays an important role in removing environmental heavy metal hazards. These Mn oxides oxidize many organic and inorganic compounds, and to scavenge a variety of other metals on their highly charged surfaces. In addition to catalyzing important process, microorganisms capable of Mn(II) oxidation are potential candidates for the removal, detoxification, and recovery of metals from the environment. Mature spores of a marine *Bacillus* sp. strain SG1 oxidize Mn(II) to MnO_2_ [95]. Vegetative cells of the same strain reduce MnO_2_ under low-oxygen conditions. The rate of MnO_2_ reduction was a function of cell density. 

Although *Bacillus* strains play a central role in biogeochemical recycling of metals in marine environment the molecular and biochemical mechanisms for most of these recycling processes are still poorly understood. It is assumed that most of the heavy metals in the environment are detoxified by reduction process. However, it is important to recognize that biochemical recycling of heavy metals is a new field and is largely limited to studies of microbial species. To remediate a range of heavy metals, it is important to select microbial strains, which carry inherently genetic machinery to reduce multiple metals. The concentration of heavy metals (Cd, Co, Cr, Hg, Pb) around hydrothermal vents is high [96]. Therefore, *Bacillus* strains living around hydrothermal vents may capable of recycling a wide range of heavy metals. The *Bacillus* strains isolated from “hydrothermal vents” should therefore be good candidates for the detoxification of environmental pollution caused by toxic heavy metals. 

## 4. Marine *Bacillus* Strains as Potential Biocontrol Agents

Most of the microorganism-based biopesticides have been developed from terrestrial bacteria. Only a few fungi have been used as efficient biocontrol agents [97]. Presently, about half of the commercially available bacterial biocontrol agents are prepared from *Bacillus* strains [12] and among them, *B. thuringiensis* accounts for more than 70% of total sales. This bacterium, omnipresent in marine environment [98], produces two proteins named Cry and Cyt during its sporulation phase, each of which is highly toxic to insects, but not to mammals or to the environment. These toxic proteins kill insects by forming pores in the gut walls. Major insect families, which can be controlled by Cry/Cyt toxins include Coleoptera, Lepidoptera, and Diptera. A wide range of plant diseases (root rot, leaf spot, anthracnose, gray mold, early blight, late blight, powdery mildew, downy mildew, and bacterial spot) can also be controlled successfully by the antagonistic *Bacillus* isolates [99]. 

The genus *Bacillus* is present in every niche of terrestrial and marine environments, even in the hot springs [100]. Many *Bacillus* species can also be found in both terrestrial and marine environments. This bacterial genus could be considered as one of the major sources of potential microbial biopesticides because of its broad genetic biodiversity [22,36], its large body of literary evidence, as well as generally regarded as safe by the US Food and Drug Administration (USFDA) and capable of withstanding unfavorable conditions through the formation of resistant spores. The main specific mechanisms involved in biocontrol of plant diseases by this bacterial genus include: competition for ecological niche/substrate in the rhizosphere, production of inhibitory chemicals, and induction of so-called systemic resistance in host plants. Marine *Bacilli* appear to be more effective biocontrol agents, compared to their terrestrial counterparts [101]. 

And so, the question remains as to whether marine or terrestrial *Bacillus* strains will be better candidates as biocontrol agents. Marine environments (with a wide variation in temperature, pressure, salt concentration and pH) are different from the terrestrial ones. The active strain, with high resistance to salt, heat, pH and stress, can be used to prepare a biological pesticide. The marine strains carry these properties naturally [15]. Marine *Bacilli* forms resistant spores quickly in unfavorable conditions, and is easily converted to powder formulations, having longer shelf-life when compared to other products containing living organisms, and can be prepared commercially at a relatively low cost (relatively unspecialized culture procedures). However, these products have some limitations due to partial protection against pathogen and pests, inconsistent effects, and a lack of ecological knowledge, which warrants precaution in field applications. There are several approaches towards the improvement of biopesticide efficacy, including selection of suitable strains, combination of synergistic strains, combinations with other forms of biopesticides, chemical pesticides, plant fertilization and agricultural practices, as well as suitable formulation and application methods [11,12].

## 5. Marine *Bacillus* Isolates as a Potential Source of Natural Carotenoids

Carotenoids are yellow, orange, and red pigments, which are widely distributed in plants and microorganisms (photosynthetic organisms, bacteria, and fungi). Chemically carotenoids usually contain a polyene C_40_ carbon skeleton, and can be acyclic, or cyclic groups, may be present at one or both ends of the backbone and having many derivatives [102]. Plants, algae, and fungi produce carotenoids containing C_40_ carbon backbone, whereas bacteria can produce a diverse range of carotenoids with either C_40_ or C_30_ carbon backbone. Each double bond in the polyene chain of a carotenoid may exist in two forms: *trans* or *cis*. Carotenoids obtained from natural sources are predominantly or entirely in *trans* form. Carotenoids are widely used as antioxidants, pro-vitamin A, and food and feed additives. Presently, synthetic carotenoids are meeting the bulk of market demands, but due to the “green wave”, coined to represent changes in consumers, industries are now looking for natural sources of carotenoids. Currently, there are no feasible sources of natural carotenoids including widely screened terrestrial microgranisns [102]. The recent screening of marine bacteria indicated that they are a promising source of natural carotenoids [103]. So far, more than 600 different carotenoids have been identified from natural sources, of which only 24 are available in human foodstuffs. The most widely used carotenoids as food are β-carotene, β-cryptoxanthin, lycopene, lutein, and violaxanthin. Carotenoids contain isoprene skeleton and are biosynthesized by tail-to-tail linkage of two C_20_ geranylgeranyl diphosphate molecules.

**Figure 24:**
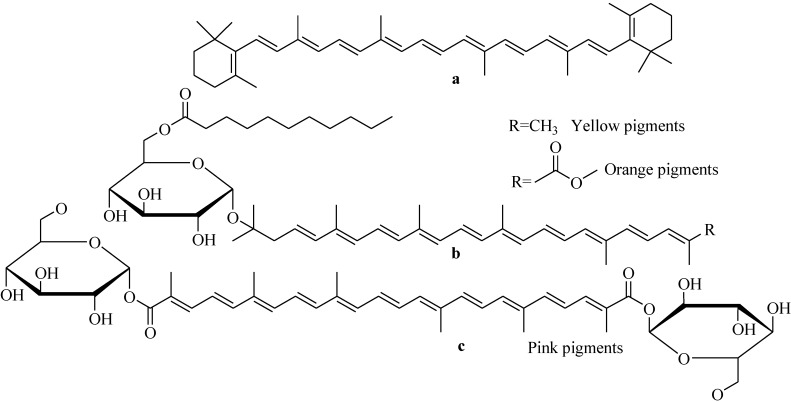
Structures of β-carotene (**a**) and main carotenoids synthesised by (**b**) HU36 and (**c**) GB1 spore-forming strains.

Irrespective of their sources, the effectiveness of carotenoinds largely depends on their bioavailablity. Due to unusual chemical structures of bacterial carotenoids, questions remained to be address bioavailability of these molecules in humans. Glycosylated carotenoids (Figure 24) isolated from marine spore-forming strains, *B. indicus* HU36 and *B. firmus* GB1, showed better bioavailability (about 4.5 times as high) than that of pure β-carotene *in vitro* digestion experiments [104]. All the marine isolates are not able to produce carotenoids. Marine yellow and orange spore-forming *Bacillus* strains have been shown to produce carotenoinds [105]. Photosynthetic marine organisms (algae and plants), either independently or in symbiosis with microorganisms, produce carotenoids to prevent oxidation by sunlight [106]. Marine yellow, red, and orange spore-forming *Bacillus* strains as well as strains living in photosynthetic organisms may be a good source of carotenoids useful for human.

## 6. Conclusions and Future Perspectives

Marine *Bacillus* species represent a rich source of structurally diverse classes of secondary metabolites including lipopeptides, polypeptides, macrolactones, fatty acids, polyketides, lipoamides, isocoumarins, and carotenoids. These structurally versatile natural products of marine isolates are derived from complex biosynthetic pathways. Some of these bioactive compounds have high potentials for the development of effective pharmaceutical and agrochemical products. Due to having genetic capability to adapt extreme conditions, *Bacillus* strains isolated from unique niches of environments (e.g., hydrothermal vent, deep sea, pH > 9.0 and salt lakes) may produce useful bioactive compounds [8]. The silent cryptic biosynthetic gene clusters of marine isolates may be activated with a view to discover new natural products by culturing them under varying stressful conditions (e.g., nutrient, pH, salinity or temperature stresses). Another important feature of the genus *Bacillus* is that they can detoxify heavy metals through reduction processes, which might be considered as candidates for the bioremediation of heavy metal toxicity. Bioremediation is an eco-friendly and cost-effective strategy for eliminating xenobiotic compounds from polluted environments. Next-generation sequencing is providing crucial insights in the molecular and biological mechanisms involved in bioremediation of environmental pollutants like heavy metal contaminations. These insights will improve bacterial bioremediation strategies, monitoring their progress, and determining their success [107]. *Bacillus*-based biopesticides can improve plant health through unique modes of action, and thus, have a high potential for commercial applications. The frequent occurrence of *B. subtilis* in the natural environment and the production capability of a vast array of antibiotics must be considered for application in pest management. Marine *Bacillus*-based biopesticides have a great potential in sustainable agricultural practices in the future. To meet the consumers demand for fully natural practices and reduced environmental hazard, marine yellow and orange spore-forming *Bacillus* strains, as well as those which are symbiotic with photosynthetic marine organisms may be a good source of natural carotenoids.
